# Exploring Medical Students’ Perspective on the Anatomage Three-Dimensional (3D) Virtual Dissection Table as a Tool to Enhance Anatomy Education

**DOI:** 10.7759/cureus.114426

**Published:** 2026-08-12

**Authors:** Abhilasha Maharshi, Pankaj K Singh, Prabhjot K Chhabra, Mahindra Anand

**Affiliations:** 1 Anatomy, Mahatma Gandhi Medical College and Hospital, Jaipur, IND; 2 Anatomy, Noida International Institute of Medical Sciences, Noida, IND

**Keywords:** 3d anatomy, advanced technology, anatomage, anatomy, dissection, medical curriculum, medical program, virtual table

## Abstract

Introduction: Human anatomy is a core subject in medical education. The rapid expansion of medical schools, coupled with a scarcity of cadavers, has necessitated innovative teaching methods in anatomy education. Emerging technologies enable virtual cadaver dissections through simulator software, including Zygote, Anatomage, and Sectra tables. Our study assessed the perceptions of medical students regarding the Anatomage three-dimensional (3D) virtual dissection table as a teaching tool, used alongside traditional cadaveric dissection.

Methods: A validated five-point Likert-scale questionnaire, supplemented with open-ended comments, was administered to 250 medical students (55% male, 45% female) after obtaining consent. Participants had exposure to Anatomage in gross anatomy, histology, neuroanatomy, and embryology during the first year of MBBS. Responses regarding satisfaction, preferences, and learning outcomes were analyzed descriptively, and percentages were calculated using GraphPad Prism 6 software (Dotmatics, Boston, Massachusetts).

Results: Students demonstrated a largely positive perception of the Anatomage virtual dissection table. A majority strongly agreed that Anatomage met their expectations and provided better visualization of anatomy (74.4%). Approximately 70.0% found group sessions on the Anatomage table engaging, and 72.0% reported improved understanding of spatial relationships. The use of Anatomage for virtual dissection of body systems was favorably perceived by 79.2% of students.

Conclusion: Anatomage enhances student engagement through superior 3D visualization of anatomical relationships, serving as a valuable supplement rather than a replacement for cadaveric dissection. Findings from this cohort (n=250) can strengthen generalizability and support hybrid teaching models in resource-limited settings.

## Introduction

Human anatomy is a core discipline in medical education and training [1,2]. Comprehensive anatomical knowledge is essential for competent medical practice. Anatomical knowledge is critical for the accurate diagnosis and treatment of many diseases [3]. Anatomy learning has traditionally depended on cadaveric dissection as the gold standard for teaching medical students about the intricacies of human structure [4]. This hands-on method offers students a tactile experience, a three-dimensional (3D) understanding, and respect for the human body.

However, the field of medical education has experienced significant changes in recent decades, with an upsurge in the number of medical schools worldwide and a corresponding rise in student enrollment [5]. This growth has posed considerable challenges to maintaining established pedagogical techniques [6]. The growth of technological resources in medical education, combined with the need for improved knowledge retention, necessitates that faculty adopt more innovative learning methods [7].

Numerous studies have recommended incorporating information technology into teaching to enhance students’ examination performance [8]. Recently, most medical programs have shifted from a teacher-centered to a student-centered approach to learning. The rise of technology plays a vital role in medical education [9]. The incorporation of technology into contemporary medical education, especially through virtual teaching methods such as virtual dissection, has grown rapidly. This approach provides a detailed 3D visualization of the anatomy of virtual cadavers [10,11]. 

The integration of digital technology into medical education represents a paradigm shift. Virtual dissection tables, 3D modeling software, Augmented reality applications, and other digital platforms have emerged as promising tools to complement or supplement traditional methods. Among these innovations, Anatomage has become a prominent advanced educational tool, released in 2011. The Anatomage table is a platform for presenting anatomy at life-size scale. Anatomage Inc., a California-based company, collaborated with Stanford University’s clinical anatomy department to develop this 3D teaching and diagnostic tool [12-14]. Anatomage bodies are built from frozen cadaver slices, segmented from real human cadavers donated for research. These slices underwent an intensive reconstruction process to recreate the cadaver’s premortem form in a 3D interactive digital representation of human anatomy. The Anatomage body portfolio includes male, female, geriatric, and pregnant cadavers [15-18]. It provides a life-size platform in the form of an 84-inch (86” x 34.9” x 27.8” (LxHxW) and weight: 330 lbs) multi-touch screen for the students. The students can view the specified structure at any layer, scale, or size (up to 0.5 mm), enabling them to conduct virtual dissections, manipulate 3D anatomical structures, and visualize relationships that are difficult to grasp with traditional dissection [18,19].

The system incorporates gross anatomy, cross-sectional imaging, neuroanatomy, histology, embryology, 77 prosections, real-time patient cases, quizzes, and realistic physiological simulation, offering an integrated approach to anatomical education [20,21]. Presently, many of the world's premier medical schools and institutions have adopted the Anatomage virtual dissection table.

Rationale for the study

With the expansion of digital anatomy resources, there remains a need for a thorough evaluation of how students perceive these tools when used alongside traditional methods across various anatomical disciplines. Understanding students’ perspectives is essential for optimizing curriculum design and resource allocation, particularly in institutions experiencing cadaver shortages. This study examined the experiences and perceptions of 250 medical students who used the Anatomage 3D virtual dissection table across multiple anatomical disciplines, including gross anatomy, histology, neuroanatomy, and embryology. The relatively large sample size can enhance the generalizability of findings and provides robust data to inform educational policy and practice.

Aims and objectives

The study aimed to assess medical students’ satisfaction with the Anatomage 3D virtual dissection table as a teaching tool and their preferences for its use across different anatomical disciplines.

The primary objective was to evaluate the perceived impact of Anatomage on learning outcomes and students’ interest in anatomy. The secondary objectives were to explore students’ attitudes toward a hybrid teaching approach combining Anatomage with traditional cadaveric dissection, and to identify the specific strengths and limitations of the Anatomage platform from the students’ perspective.

## Materials and methods

Study design and setting

This cross-sectional descriptive study was conducted at the Department of Anatomy, Mahatma Gandhi Medical College and Hospital, Jaipur, Rajasthan, in the year 2025. The study employed a quantitative approach, supplemented by open-ended qualitative responses, to comprehensively capture students' perception and experience.

Sample size

The study population included 250 second- and third-year MBBS students (125 from each batch) who successfully completed the anatomy course using both traditional cadaveric dissection and the Anatomage 3D virtual dissection table. The demographic breakdown was 55% male and 45% female students. All participants provided informed consent before enrollment in the study.

Ethical considerations

The study protocol was reviewed and approved by the Institutional Ethics Committee (approval number: MGMC&H/IEC/JPR/2025/5068) of our college. All participants were informed of the voluntary nature of participation, the confidentiality of responses, and their right to withdraw at any time without consequences. No identifying information was collected in the questionnaires, ensuring anonymity of responses.

Inclusion criteria

Second- and third-year medical students who completed and passed the anatomy curriculum in the first MBBS had exposure to both Anatomage and traditional cadaveric dissection and gave voluntary consent to participate in the study.

Exclusion criteria

Students who had not completed full anatomy curriculum were not included in the study. Students who declined to provide informed consent and those who submitted incomplete questionnaire responses were excluded from the study.

Before data collection, students of two academic sessions were exposed to the Anatomage 3D virtual dissection table version 12 across four anatomical disciplines by different methods of learning such as demonstrations, small group discussions, self-directed learning, quizzes, and case discussions.

Students received teaching sessions on the Anatomage table across the following anatomical disciplines: (1) Gross anatomy: Students used Anatomage to explore regional anatomy, visualize anatomical relationships in multiple planes, and perform virtual dissections, complementing physical cadaveric work. (2) Histology: The platform's digital microscopy capabilities were used to display high-resolution histological micrographs, allowing students to examine tissue architecture at various magnifications. (3) Neuroanatomy: Anatomage was used to visualize neuroanatomical structures, including the brain, spinal cord, and peripheral nervous system, in three dimensions. (4) Embryology: Different stages and anatomical changes during embryological development were demonstrated using the Anatomage table.

Data collection instrument

A validated questionnaire employing a five-point Likert scale was created in Google Forms and administered to participants. The scale ranged from 1 (Strongly Disagree) to 5 (Strongly Agree), enabling a detailed evaluation of student perceptions.

The questionnaire covered the following areas: (1) Overall satisfaction: General contentment with Anatomage as a teaching tool. (2) Discipline-specific preferences: Utility of Anatomage in gross anatomy, histology, neuroanatomy, and embryology. (3) Learning outcomes: Perceived impact on knowledge acquisition and understanding. (4) Interest and engagement: Effect on motivation and interest in anatomy. (5) Quality of visualizations: Assessment of image quality and 3D representations. (6) Integration preferences: Attitudes toward hybrid teaching models and broader integration with other teaching aids.

Additionally, open-ended questions allowed students to provide qualitative feedback on their experiences and offer suggestions for improvement.

Data analysis

Quantitative data from Likert-scale responses were analyzed descriptively using GraphPad Prism 6 software (Dotmatics, Boston, Massachusetts). Results were expressed as percentages to facilitate interpretation. Responses were categorized into positive (Agree and Strongly Agree), neutral, and negative (Disagree and Strongly Disagree) groupings for certain analyses. Descriptive statistics in the form of percentages were calculated and recorded using Microsoft Office Excel 2021 (Microsoft Corp., Redmond, WA, USA). Qualitative data from open-ended responses were analyzed thematically to identify common patterns, concerns, and suggestions.

## Results

A total of 250 MBBS students participated in the study, representing a 100% response rate from eligible students. The gender distribution of the respondents indicates a slightly higher proportion of male participants (54.8%) than female participants (45.2%) (Table 1).

**Table 1:** Gender distribution

Gender	Frequency	Percent
Female	113	45.2%
Male	137	54.8%
Total	250	100.0%

Most respondents were in the 18-20 years age group (52.8%), followed by those aged 21-23 years (44.4%). A small proportion of students were above 23 years of age (Table 2).

**Table 2:** Age distribution

Age group	Frequency	Percent
18-20	132	52.8%
21-23	111	44.4%
24-26	5	2.0%
27+	2	.8%
Total	250	100.0%

**Figure 1:**
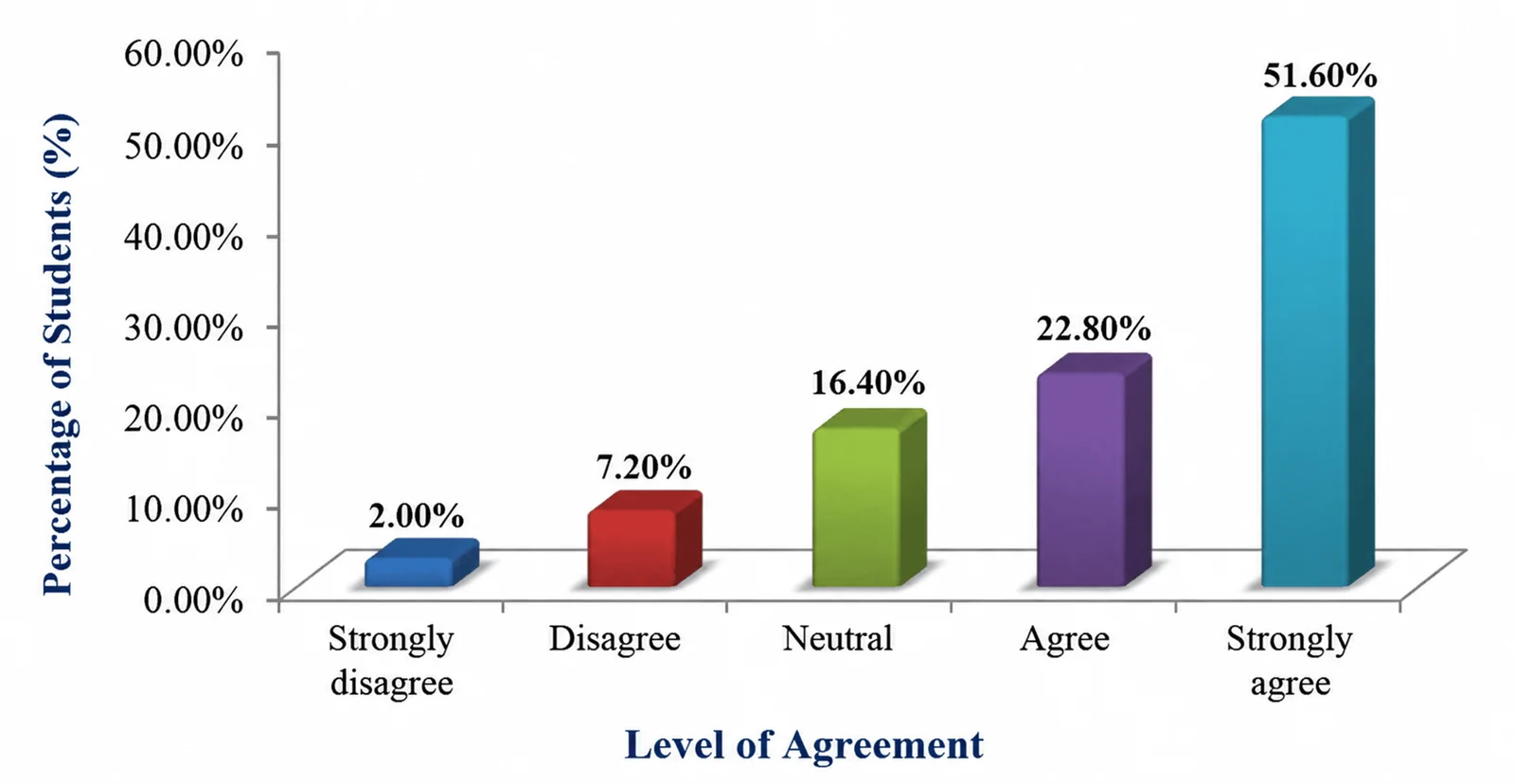
Students' perception of the Anatomage table for better visualization of anatomical structures

A strong positive perception was observed, as 77.6% of respondents agreed that Anatomage met their expectations as an innovative teaching tool. Only a small portion disagreed 11.2%, and 11.2% were neutral. This suggests that students generally perceive Anatomage as meeting its intended purpose of modernizing anatomy instruction and offering value beyond traditional teaching aids. The results show very strong agreement that Anatomage enhances visualization (Figure 1): 51.6% strongly agreed and 22.8% agreed, for a total of 74.4% positive responses. This indicates that Anatomage’s 3D and layered visualization capabilities are widely appreciated, helping learners “see” anatomical structures more clearly than with some conventional methods.

Approximately 72% of respondents agreed that Anatomage improved their understanding of spatial relationships, indicating that Anatomage is valuable in helping students understand how structures relate in 3D space, which is an important aspect of anatomy that students often struggle with when relying only on two-dimensional diagrams or static images (Table 3).

**Table 3:** Students’ perception on Anatomage gives better visualization of anatomy

Student’s response	Frequency	Percent
Strongly disagree	9	3.6%
Disagree	15	6.0%
Neutral	46	18.4%
Agree	68	27.2%
Strongly agree	112	44.8%
Total	250	100.0%

As per 73.6% of respondents, Anatomage is effective for virtual dissection of body systems. A strong majority (79.6%) said that Anatomage addresses cadaver availability limitations. Therefore, students recognize Anatomage as a practical solution in settings where cadavers are limited due to cost, ethical, supply, or storage constraints, while still supporting continuity in anatomical learning and practice and exploring structures that may be difficult to visualize in cadaver labs.

**Figure 2:**
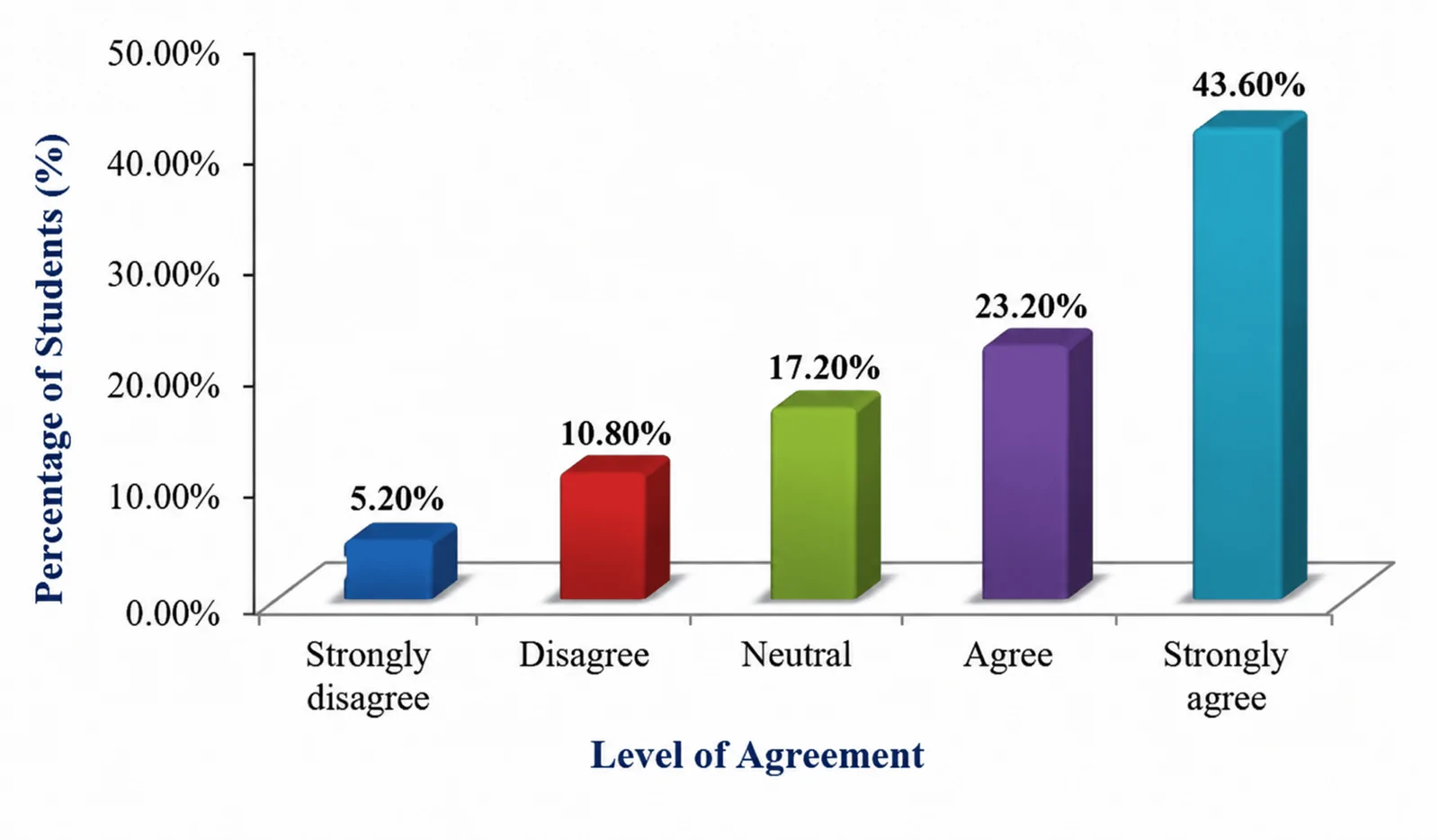
Students' perception of the Anatomage table in preparing them for clinical practice

Most students reported benefits in memory and recall, with 63.6% agreeing, suggesting that Anatomage likely supports memorization through visual reinforcement, interactivity, and repeated viewing, though some students may still rely more on cadaver exposure, textbooks, or traditional repetition strategies for retention. About 66.8% of respondents agreed (Figure 2) that Anatomage prepares them better for clinical practice. This suggests that many students see clinical relevance in Anatomage, perhaps because it mirrors imaging-style visualization and helps connect anatomical concepts to clinical interpretation, even though some still feel cadaver-based learning may be more clinically authentic.

A very high proportion (78.8%) agreed that Anatomage is a good supplement to cadaver dissection, while only 14.4% disagreed and 6.8% were neutral. A clear majority (72.4%) reported that Anatomage is helpful for gross anatomy learning. This indicates that students generally find it effective for studying large structures, organ systems, and relationships between parts, likely due to the ability to rotate, isolate, and virtually “cut” structures for better understanding. About 58.8% agreed that Anatomage displays histology micrographs better. This suggests Anatomage may be beneficial for histology, especially for visibility and shared learning, but a sizeable group may still prefer microscopes due to realism, focus control, or familiarity with traditional slide-based practice. A strong majority (69.6%) viewed it as effective for neuroanatomy teaching. Neuroanatomy is often considered complex, so the high agreement implies that Anatomage’s 3D visualization is particularly helpful for understanding brain structures, pathways, and spatial orientation that are difficult to grasp with a flat image. More than half of respondents (57.6%) stated that Anatomage provides realistic embryological representation. This indicates general support but also mixed perceptions, suggesting that embryology visualization may work well for many learners, although some may feel the representations are not detailed enough or prefer models, charts, or videos specifically designed for embryological development.

**Figure 3:**
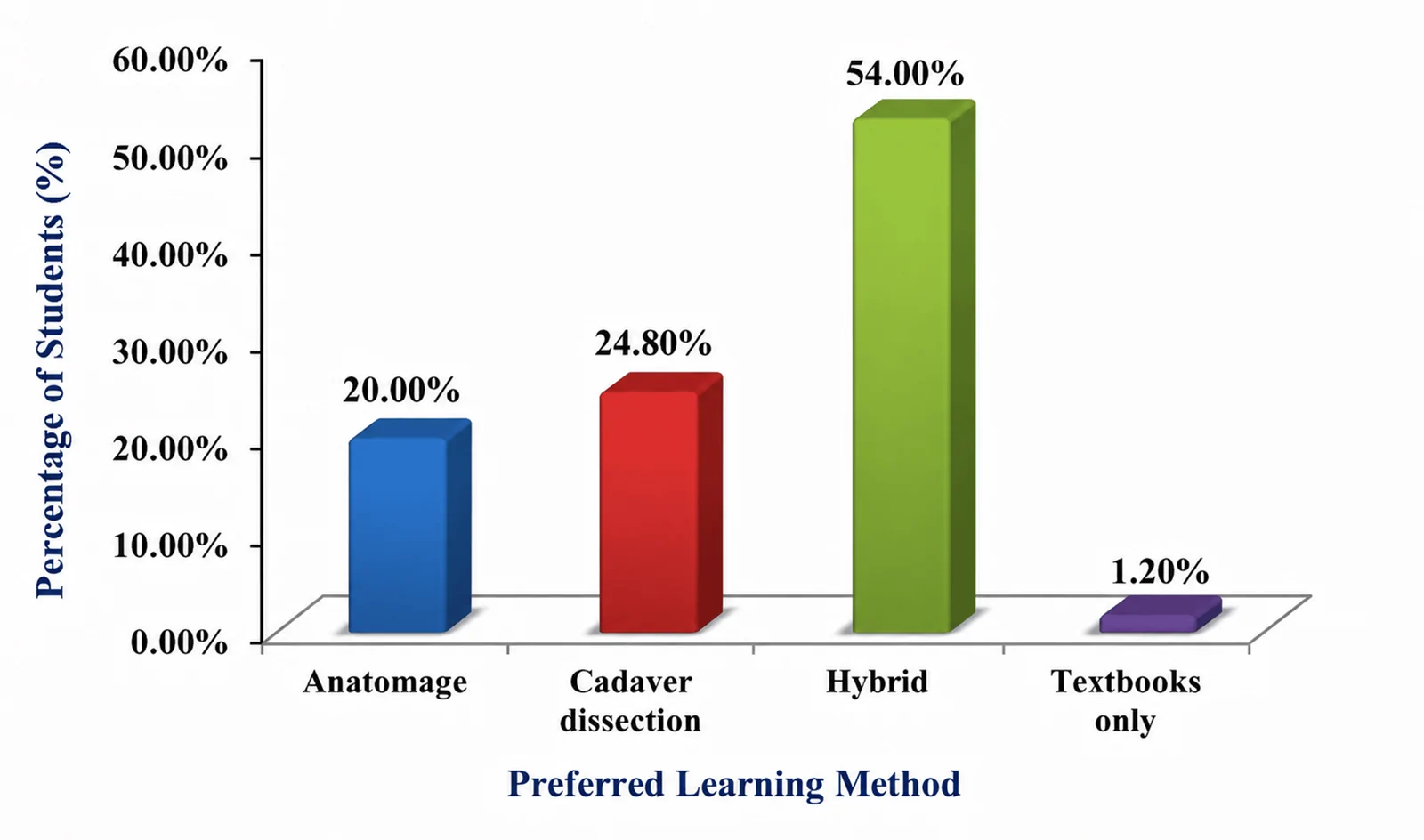
Students' perception on preferred method of learning

The most preferred approach was hybrid learning (54%), combining Anatomage and cadaver dissection. Cadaver-only learning (24.8%) was the next most preferred, followed by Anatomage-only (20%), and very few preferred textbooks alone (1.2%) (Figure 3). This clearly indicates that students value a blended approach, suggesting they want the realism of cadavers alongside the clarity and repeatability of Anatomage for deeper understanding.

**Figure 4:**
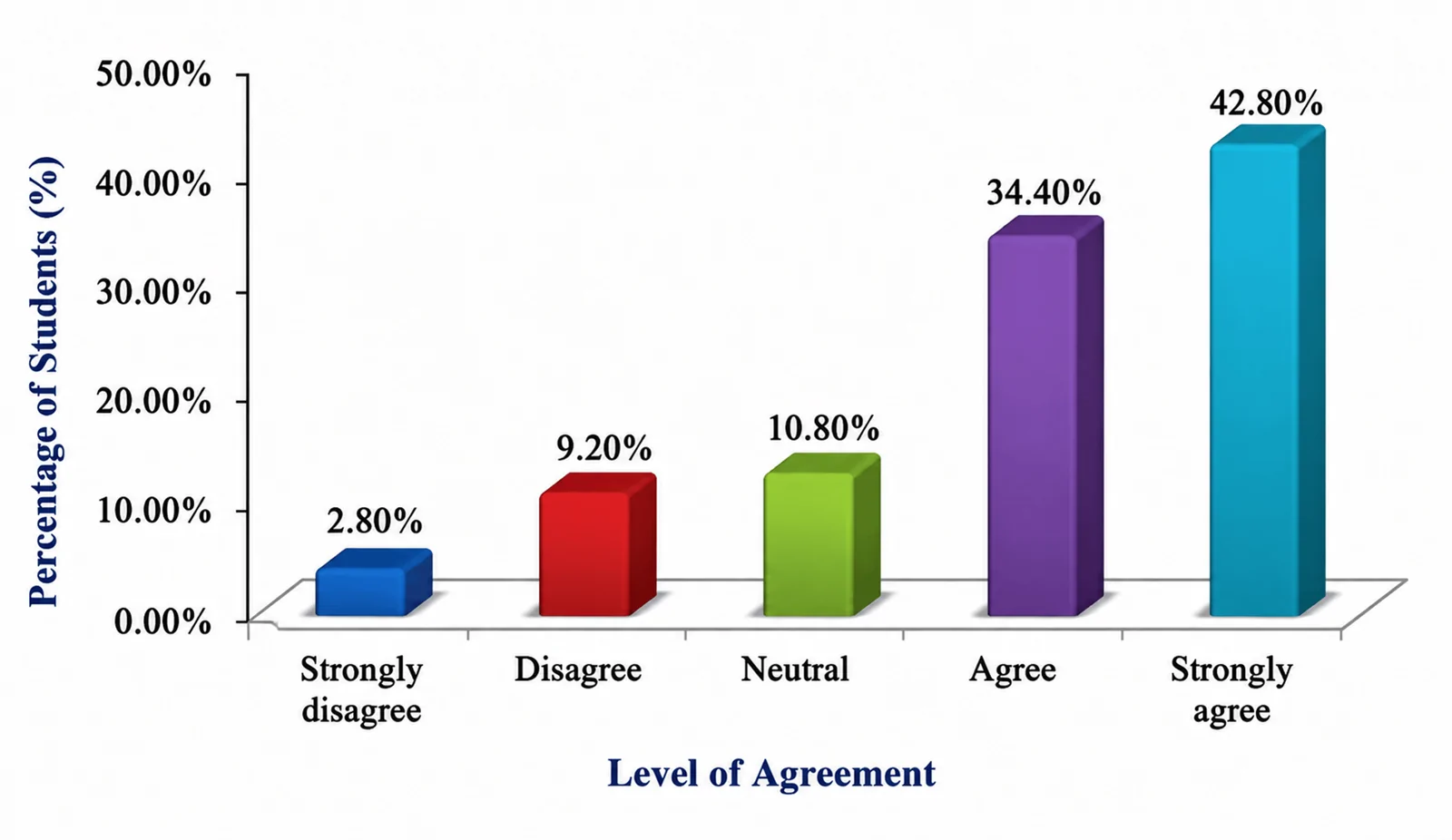
Students' perception of the Anatomage table in integrating classroom and laboratory learning

A positive trend appears here: 68.8% agreed that Anatomage is a better option for setting quizzes and exam questions. This suggests students believe Anatomage can enhance assessment quality by enabling 3D identification questions and applied anatomy tasks, though some may still prefer conventional written or cadaver-based assessment formats. A large majority (77.2%) agreed that Anatomage integrates classroom and laboratory learning. This implies that the tool may help bridge theoretical lectures with practical understanding by allowing students to explore anatomical content immediately in a lab-like interactive environment, improving continuity in learning (Figure 4).

**Figure 5:**
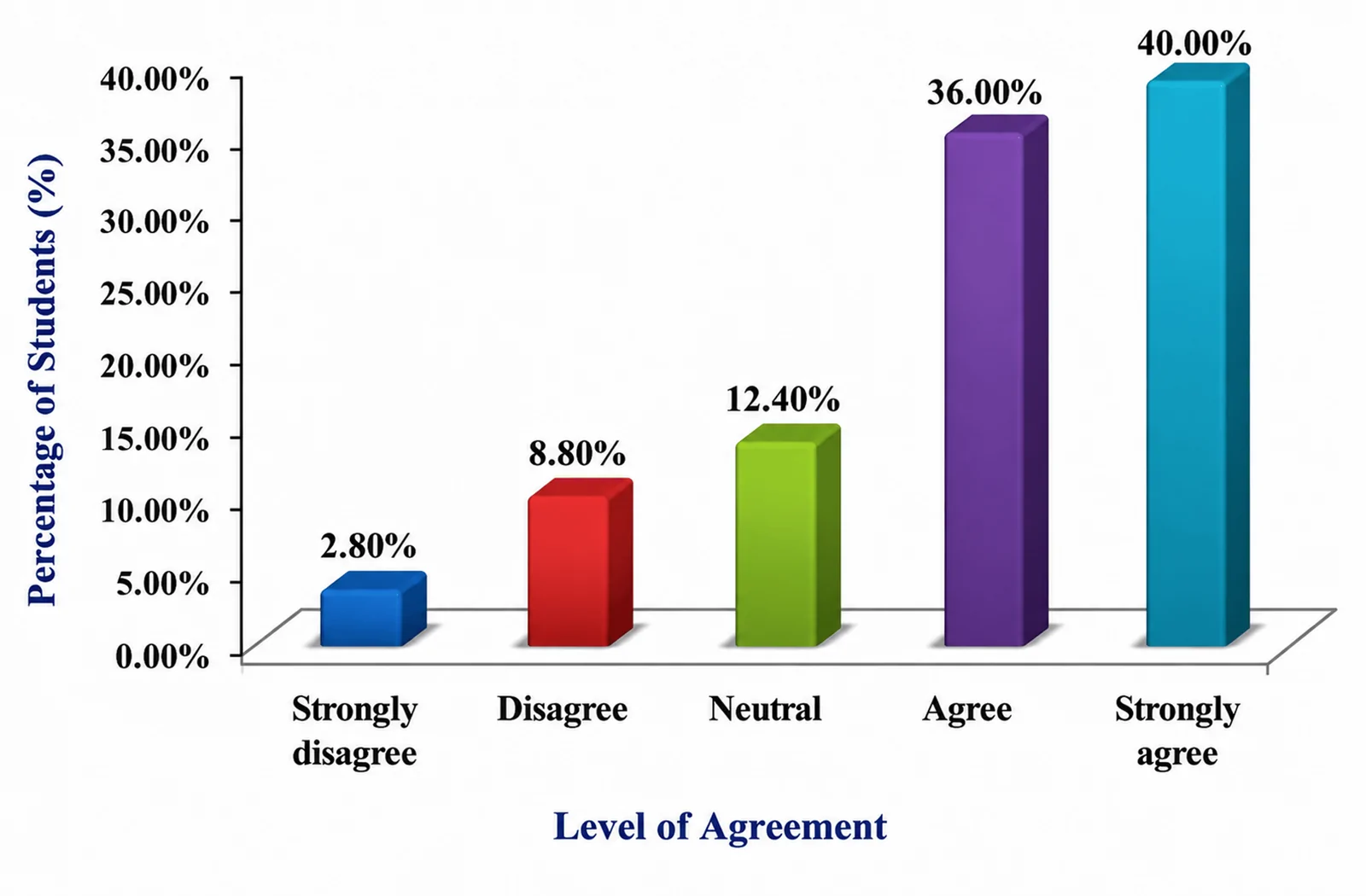
Students' perception of the Anatomage table in achieving intended learning outcomes

Overall, 70% stated that Anatomage positively influenced their learning experience. About 76% of respondents agreed that Anatomage achieves the intended learning outcomes (Figure 5). This suggests that the majority believe the tool contributes meaningfully to learning objectives such as visualization, understanding, and retention-indicating perceived educational effectiveness from the learner’s perspective.

A very strong endorsement was reported, with 80% agreeing or strongly agreeing that Anatomage should be adopted as a standard teaching tool. Overall, this shows that students widely support the broader integration of Anatomage into the curriculum, likely because of its perceived benefits for understanding and engagement.

## Discussion

Cadaver dissection has proven to be the ideal method for learning anatomy. However, technological advancements have revolutionized the visualization and dissection of anatomical structures. This transformation is particularly evident in the realm of the Anatomage 3D virtual dissection table [22]. The Anatomage virtual dissection table can be used alongside cadavers or as a standalone tool for teaching anatomy [22,23].

This study examined medical students' perceptions of the Anatomage virtual dissection table for learning human anatomy at the Department of Anatomy, Mahatma Gandhi Medical College, Jaipur, in 2025. A total of 250 students participated, with 55% male and 45% female students. In this group, 77.6% of students (36.8% agree, 40.8% strongly agree) reported that the Anatomage table meets their expectations as an innovative teaching tool. Brown et al. reported that most students responded positively to the involvement of Anatomage tables as an effective learning tool [24].

Visualization and spatial understanding

Regarding visualization, our findings indicate that 74.4% of respondents agreed that the use of Anatomage provides better visualization of anatomy. Similarly, 72% stated that Anatomage enhanced their understanding of spatial relationships. Likewise, Kar et al. revealed in their study that more than 65% believed that virtual tables facilitate the understanding of anatomical structures due to their high resolution [25]. Alasmari et al. reported that most medical students agreed that the Anatomage table improved their ability to study various anatomical structures [17]. She also reported that most students believed that visualizing the digital human body is easier with Anatomage because of its ability to rotate images along multiple axes.

Role as a supplement to cadaver dissection and integration into the curriculum

In the present study, 78.8% of students agreed that the Anatomage table is a valuable supplement to cadaver dissection in the anatomy laboratory. This finding aligns with preference data showing that 54% of students explicitly preferred a hybrid method, suggesting that Anatomage is seen not only as a replacement but also as a complementary tool that enhances traditional dissection by providing clarity and broader exposure.

Furthermore, 80% of respondents stated that Anatomage should be adopted as a standard teaching tool in the anatomy curriculum, and 79.6% agreed that it effectively integrates classroom and laboratory learning, strongly supporting its inclusion in the formal curriculum. Chytas et al. recommended virtual dissection and the Anatomage as additional resources for learning anatomy [26]. Similarly, Niedermair et al. noted that students consider virtual techniques as an adjunct rather than a replacement for dissection-based anatomy [27]. Alasmari et al. found that over 80% of participating students favored using Anatomage alongside cadavers to gain a better understanding of human anatomy [17].

Discipline-specific utility

When asked which anatomical region is best visualized, 46.8% of students chose gross anatomy, 32.4% selected neuroanatomy, and smaller percentages chose embryology (5.2%), histology (8.8%), or all regions equally (2%). These distributions suggest that students perceive the greatest added value of Anatomage in large-scale, structurally complex regions where 3D understanding is essential, especially gross anatomy. Kažoka et al. observed that the Anatomage table enhanced visualization and memorization of different anatomical organs [28]. Anand and Singel [16] compared learning neuroanatomy with Anatomage to dissection. This study reported no statistically significant differences between the two methods of learning anatomy.

Impact on learning outcomes and assessment

In the present study, 74% of students reported that they achieved positive learning outcomes. It is important to note that this study measured perception rather than objective learning outcomes. Also, 63.6% of students agreed that using Anatomage helps in memorization of anatomical details. In the study conducted by Sultana et al., students who were exposed to the virtual dissection table scored comparatively better on post-tests than those exposed to cadaveric dissection [29].

Further, 66.8% of students felt that it helps them to prepare for clinical practice, reflecting their perception that the integration of 3D visualization supports a clinically oriented understanding. In terms of assessment, 68.8% of students agreed that using Anatomage to set quizzes and examination questions is a better option.

Influence on classroom experience and addressing resource limitations

The current study revealed that 70% of students had a positive classroom experience of Anatomage, indicating that most students experienced enhanced engagement during sessions. Also, 79.6% of students agreed that Anatomage addresses limitations in anatomy education arising from restricted cadaver availability and large student cohorts. Brown et al. conducted a cross-sectional study revealing that more than 75% of students reported that Anatomage made learning more engaging. More than 60% of students in their study were of the opinion that it improved 3D anatomy understanding [24].

Pedagogical implications

The findings of this study suggest several implications for anatomy education: (1) Curriculum design: Anatomy curricula should intentionally incorporate digital technologies as complementary rather than replacement tools, with clear learning objectives for each modality. (2) Resource allocation: Institutions should consider investments in digital platforms such as Anatomage, particularly where cadaver access is limited, while maintaining commitment to traditional methods where feasible. (3) Faculty development: Educators need training to effectively integrate digital technologies into their teaching, maximizing educational value and avoiding redundancy. (4) Student preparation: Pre-dissection virtual sessions can enhance the efficiency and effectiveness of cadaveric work by familiarizing students with anatomical relationships before hands-on experience. (5) Assessment: Assessment methods should evolve to evaluate competencies through both traditional and digital modalities, including 3D spatial reasoning and cross-sectional anatomy interpretation.

## Conclusions

This study demonstrates that the Anatomage 3D virtual dissection table is perceived as a valuable educational tool in anatomy education. The technology shows overwhelming students' response to hybrid teaching models, combining Anatomage with traditional cadaveric dissection. The findings support integrating digital technologies into the anatomy curriculum as complementary tools that enhance, rather than replace, traditional methods. This study emphasizes that the future of anatomy education lies not only in choosing between traditional and digital methods but also in strategically uniting them to create an educational experience that is more effective, accessible, and engaging than either approach alone. This study contributes to the evidence base for such hybrid models and provides a foundation for continued innovation in medical education.
